# Estimation of the need for bilateral intravitreal anti-VEGF injections in clinical practice

**DOI:** 10.1186/s12886-016-0317-y

**Published:** 2016-08-09

**Authors:** Audrey Giocanti-Auregan, Ramin Tadayoni, Typhaine Grenet, Franck Fajnkuchen, Sylvia Nghiem-Buffet, Corinne Delahaye-Mazza, Gabriel Quentel, Salomon Y. Cohen

**Affiliations:** 1 Department of Ophthalmology, Hôpital Avicenne, AP-HP and University Paris 13-Bobigny, Seine Saint Denis, France; 2 Departement Hospitalo-Universitaire Vision et Handicaps, Paris, France; 3 Department of Ophthalmology, Hôpital Lariboisière, AP-HP and University Paris 7, Paris, France; 4 Centre Ophtalmologique d’Imagerie et de Laser, 11 rue Antoine Bourdelle, 75015 Paris, France; 5 Hôpital Intercommunal and University Paris-Est-Creteil, Creteil, France

**Keywords:** Intravitreal injection, Age-related macular degeneration, Anti-VEGF, Diabetic macular edema, Retinal diseases

## Abstract

**Background:**

To estimate the need for bilateral intravitreal anti-VEGF injections in patients treated for neovascular age-related macular degeneration (nAMD), diabetic macular edema (DME), retinal vein occlusion, choroidal neovascularization (CNV) in high myopia, and other causes of CNV.

**Methods:**

All consecutive patients treated with intravitreal anti-VEGF injection over a 1-month period were included in a prospective multicenter survey. The reason for intravitreal anti-VEGF injection and the involvement of the fellow eye in the pathology requiring a treatment with intravitreal anti-VEGF were recorded. A time interval between bilateral injections longer than 1 month, within a 1-month period, and same-day bilateral injections were recorded.

**Results:**

A total of 1335 patients were included, corresponding to 1024 (76.7 %) patients treated for nAMD, 167 (12.5 %) for DME, and 144 (10.8 %) for other reasons. Four hundred and fifty-nine (34.4 %) patients were treated bilaterally with a time interval between injections longer than 1 month, 170 (12.7 %) were treated bilaterally within a 1-month interval, and 87 (6.6 %) had same-day bilateral injections. Bilateral injections were more frequent in diabetic patients than in nAMD patients (respectively 48 % vs. 36 %, *p* = 0.0033).

**Conclusions:**

Patients with DME are more likely to be treated bilaterally with anti-VEGF injections. As the rate of second eye involvement requiring treatment increases progressively over time, a same-day bilateral injection strategy will become more common as it decreases the administrative burden on the healthcare system and treatment burden experienced by patients.

**Electronic supplementary material:**

The online version of this article (doi:10.1186/s12886-016-0317-y) contains supplementary material, which is available to authorized users.

## Background

The number of intravitreal anti-VEGF injections has increased progressively, partly because of the extension of anti-VEGF indications to various chorioretinal diseases. Anti-VEGFs have been first indicated for the treatment of neovascular age-related macular degeneration (nAMD) [1, 2], then macular edema complicating retinal vein occlusion (RVO) [3–6], diabetic macular edema (DME) [7–9], and other causes of choroidal neovascularization (CNV) such as high myopia.

nAMD and DME frequently affect both eyes [10, 11]. The fellow eye involvement, which is often the better-seeing eye, is likely to have a significant impact on patient quality of life.

Patients with nAMD require anti-VEGF treatment in their fellow eye in about 7.5 % of cases each year [12]. However, this rate may be different from one country to another, most often because of different criteria for health insurance system coverage. In France, the rate of bilateral injections in retinal pathologies is unknown and the use of bevacizumab was off-label regardless of the indication, during the duration of the survey. Ranibizumab has been authorized for the treatment of nAMD, DME, macular edema complicating RVO, CNV complicating pathologic myopia and pseudoxanthoma elasticum and its use is covered by the health insurance system. Aflibercept is authorized for the treatment of nAMD, macular edema complicating central RVO and DME. Thus, evaluating the burden of intravitreal injections on French patients is pertinent there is no administrative limitation, because the number of intravitreal injections is not regulated by the healthcare system but only by the need for treatment. Administrating bilateral same-day intravitreal injections is becoming more common. Indeed, a US survey has shown that 46 % of retina specialists administered bilateral same-day intravitreal injections [13] limiting the frequent need to access the clinic.

The aim of this study was to estimate the need for bilateral intravitreal anti-VEGF injections in a real-life setting in patients treated for nAMD, DME, RVO, myopic CNV, and other causes of CNV.

## Methods

All consecutive patients who received an intravitreal injection of anti-VEGF over a 1-month period (July 1 to 31, 2014) were included in a prospective multicenter study. Three centers participated in the study: a private practice in Paris (Centre d’Imagerie et de Laser), and two public and academic ophthalmological departments, one in a Paris hospital (Lariboisière hospital) and one in a hospital in Paris suburbs (Avicenne hospital). The private practice is one of the center performing the largest number of intravitreal injections in France and both academic hospitals selected are specialized in DME. The 3 centers are located in a same geographic area. This study was conducted in accordance with the tenets of the Declaration of Helsinki, and an informed written consent was obtained from subjects. Approval was obtained from the France Macula Federation ethical committee (committee’s reference number: FMF 2015–137).

All data were collected in each center by one of the authors (AGA) and included: the reason for intravitreal injection of anti-VEGF and the involvement of the fellow eye in the pathology requiring an intravitreal injection of anti-VEGF. The number of patients requiring bilateral treatment previously (at any time), within a 1-month interval, and receiving same-day intravitreal injections was assessed.

Patients were included regardless of the anti-VEGF drug used. Patients treated with other intravitreal treatments (triamcinolone, dexamethasone implant) were excluded. Patients injected twice during the study period (once in one eye, and once in the fellow eye) were only taken into account once. Sub-group analysis was conducted in patients treated for DME or nAMD in order to compare the rate of bilateral treatment. A second sub-analysis was conducted to compare the time interval between bilateral injections in patients treated for nAMD and DME.

For statistical analysis, Graphpad prism software was used and a Chi2 test was performed.

The survey was conducted in accordance with the tenets of the Declaration of Helsinki, and an informed consent was obtained from all patients.

## Results

A total of 1335 patients from three ophthalmological centers were included over a 1-month study period. They received a total of 1501 intravitreal anti-VEGF injections. Among them, 1024 (76.7 %) patients were treated for nAMD and 167 (12.5 %) for DME. The details of the pathologies and the distribution between the three centers are presented in Table 1. All the data collected are available in the Additional file 1: Table S1.

**Table 1:** Distribution of the different pathologies requiring anti-VEGF treatment between the three centers

Number of patients	Center 1 (%)	Center 2 (%)	Center 3 (%)	Overall results (%)
nAMD	674 (86.6)	269 (66.1)	81 (54)	1024 (76.7)
DME	17 (2.2)	98 (24.1)	52 (34.7)	167 (12.5)
Vein occlusions	44 (5.7)	26 (6.4)	11 (7.3)	81 (6.1)
CNV in high myopic eyes	22 (2.8)	3 (0.7)	6 (4)	31 (2.3)
Others	21 (2.7)	11 (2.7)	0	33 (2.4)
Total	778 (58.3)	407 (30.5)	150 (11.2)	1335 (100)

Raw numbers (percentage)

*CNV* choroidal neovascularization, *DME* diabetic macular edema, *nAMD* neovascular age-related macular degeneration

Four hundred and fifty-nine (34.4 %) patients were treated bilaterally with a time interval between injections longer than 1 month, 170 (12.7 %) were treated bilaterally within a 1-month period, and 87 (6.6 %) had same-day bilateral injections.

**Fig. 1:**
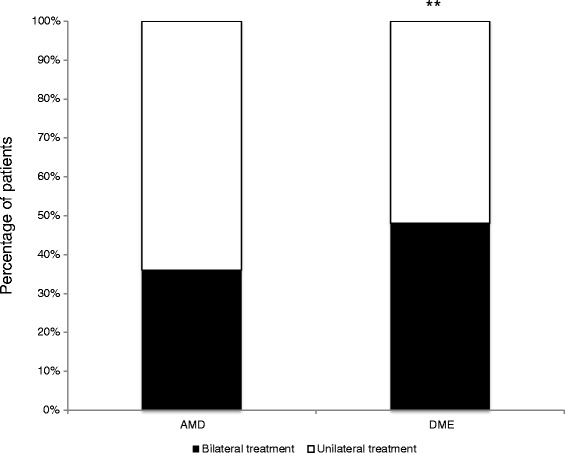
Percentage of patients who received bilateral intravitreal anti-VEGF injections regardless of the time interval between bilateral injections, showing a significant difference between nAMD and DME (respectively 36 % vs. 48 % of patients, *p* <0.01)

A sub-group analysis was performed in patients treated for nAMD and DME in order to compare the rate of bilateral treatment. The results are presented in Fig. 1. The rate of bilateral injections was significantly higher in diabetic patients than in nAMD patients (respectively 48 % vs. 36 %, *p* = 0.0033).

**Fig. 2:**
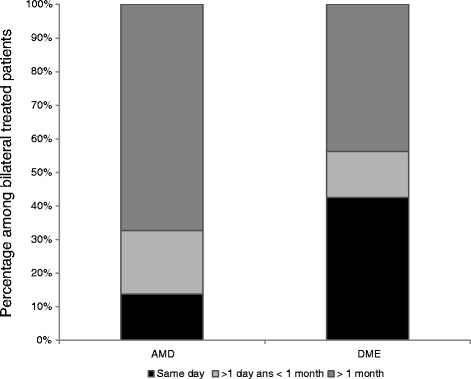
Distribution of the time interval between bilateral injections in nAMD and DME patients. Same-day bilateral injections were significantly more common in DME patients than in nAMD patients (respectively 42.5 % vs. 13.8 %, *p* <0.001)

The time interval between bilateral injections was then analyzed in patients treated for nAMD and DME (Fig. 2). In nAMD patients, bilateral injections were administered on the same day, within a time interval >1 day and < 1 month, and within a time interval longer than 1 month respectively in 13.8, 19, and 67.2 % of cases. In DME patients, the distribution was respectively in 42.5, 13.7 and 43.8 % of cases. The number of cases receiving same-day bilateral treatment was significantly higher in DME patients than in nAMD patients (respectively 42.5 % vs. 13.8 %, *p* <0.001). In vein occlusion, bilateral injections were administered in 2 cases (2.5 %) only, within a time interval longer than 1 month for both. In high myopia, none of the patient received same-day bilateral injection, 1 patient (3.2 %) received bilateral treatment within a time interval >1 day and < 1 month, and 5 (6.2 %) within a time interval longer than 1 month. No case of endophthalmitis was reported.

## Discussion

In this study, we estimated the proportion of patients treated for ocular pathologies with anti-VEGF therapy and needing bilateral injections over a 1-month period. Our data show that the rate of bilateral injections was of 36 % in nAMD. Only a few studies have investigated the rate of bilateral injections in nAMD although a bilateral involvement in this disease of about 5–20 % per year has been previously reported [14–19]. In addition, Zarranz-Ventura et al. [12], have reported an incidence of second eyes treated with ranibizumab for nAMD of about 7.5 % per year which increased to 14 % per year when patients with visual acuity lower than 20/200 in the fellow eye were excluded. Compared to nAMD, the proportion of bilateral involvement in DME is higher and estimated to range between 60 and 80 % [20–22]. Consistently, a significantly higher (48 %, *p* = 0.0033) proportion of bilateral injections in DME patients was found in our study. This high rate of bilateral involvement in DME could be due to the systemic nature of disease, making it more likely to affect both eyes. The discrepancy between the reported rate of bilateral injections in this study and that described in nAMD and DME could be explained by the design of our study. Indeed, the rate of bilateral involvement increases with the follow-up and disease duration and in this study, it was assessed at a specific time point (i.e., within a 1-month period).

The time interval between bilateral injections was also evaluated in this study. In the management of nAMD patients, there was a tendency to first administer injection in one eye then followed a few days to a few weeks later by an injection in the fellow eye. Therefore, only 13.8 % of patients received same-day bilateral injections. By contrast, the treatment was more often concomitant in the management of DME patients as 42.5 % of patients in our study received same-day bilateral injections. Two parameters could have influenced this proportion. First, patient preference could have contributed because one visit for same-day bilateral injections decreases the burden on patients and their families compared to two separate visits. Second, bilateral treatment feasibility requires that both eyes are perfectly synchronized and have the same reactivation time.

Bilateral injections performed the same day when indicated, always according to the patient preference, may limit the frequent visits to the clinic for the patient and his accompanying persons. When administering same-day bilateral intravitreal injections, local and systemic complications should be considered. Local complications of intravitreal injections include raised intraocular pressure, uveitis, retinal detachment and subretinal hemorrhages [23]. Retrospective studies have investigated the safety of same-day bilateral intravitreal injections and shown that when separate povidone-iodine preparations, speculum, needles, and syringes are used for each eye, injections are well tolerated [24, 25]. In the review by Lima et al. [26], it has been suggested that the ocular complications (endophthalmitis and intraocular inflammation) related to same-day bilateral intravitreal injections are similar to those appearing after unilateral injections [26]. None was reported in our study. Although rare, systemic side effects of intravitreal anti-VEGF treatment have also been reported, including myocardial infarctions, strokes and thromboembolic events [27]. However, it remains unknown whether an increase in systemic dose due to bilateral injections may increase the risk. Several studies have evaluated the outcomes and complications of same-day bilateral intravitreal injections and no major systemic events have been reported [28, 29]. The present survey was not designed to give any information concerning local or systemic tolerance of anti-VEGF drugs administrated the same day. The main limitation of our study is its descriptive nature and the limited data on patient characteristics and history of ocular diseases. However, our results provide relevant data in a real-world setting by evaluating the frequency of bilateral intravitreal anti-VEGF injections administered by French retina specialists in patients treated for retinal pathologies.

## Conclusion

Efficacy and safety outcomes of bilateral anti-VEGF injections have been recently studied.

As the rate of second eye involvement requiring treatment increases progressively over time, a same-day bilateral injection strategy will become more common as it decreases the administrative burden on the healthcare system and the treatment burden experienced by patients and families.

## Abbreviation

CNV, choroidal neovascularization; DME, diabetic macular edema; nAMD, neovascular age-related macular degeneration; RVO, retinal vein occlusion; VEGF: vascular endothelial growth factor

## Additional file

Additional file 1: Table S1. Excel table including all the raw data collected. (XLSX 217 kb)

## References

[1] Rosenfeld PJ, Brown DM, Heier JS, Boyer DS, Kaiser PK, Chung CY (2006). Ranibizumab for neovascular age-related macular degeneration. N Engl J Med.

[2] Brown DM, Kaiser PK, Michels M, Soubrane G, Heier JS, Kim RY (2006). Ranibizumab versus verteporfin for neovascular age-related macular degeneration. N Engl J Med.

[3] Heier JS, Campochiaro PA, Yau L, Li Z, Saroj N, Rubio RG (2012). Ranibizumab for macular edema due to retinal vein occlusions: long-term follow-up in the HORIZON trial. Ophthalmology.

[4] Varma R, Bressler NM, Suñer I, Lee P, Dolan CM, Ward J (2012). Improved vision-related function after ranibizumab for macular edema after retinal vein occlusion: results from the BRAVO and CRUISE trials. Ophthalmology.

[5] Campochiaro PA, Clark WL, Boyer DS, Heier JS, Brown DM, Vitti R (2015). Intravitreal Aflibercept for Macular Edema Following Branch Retinal Vein Occlusion: The 24-Week Results of the VIBRANT Study. Ophthalmology.

[6] Ogura Y, Roider J, Korobelnik J-F, Holz FG, Simader C, Schmidt-Erfurth U (2014). Intravitreal aflibercept for macular edema secondary to central retinal vein occlusion: 18-month results of the phase 3 GALILEO study. Am J Ophthalmol.

[7] Korobelnik J-F, Do DV, Schmidt-Erfurth U, Boyer DS, Holz FG, Heier JS (2014). Intravitreal aflibercept for diabetic macular edema. Ophthalmology.

[8] Schmidt-Erfurth U, Lang GE, Holz FG, Schlingemann RO, Lanzetta P, Massin P (2014). Three-year outcomes of individualized ranibizumab treatment in patients with diabetic macular edema: the RESTORE extension study. Ophthalmology.

[9] Brown DM, Nguyen QD, Marcus DM, Boyer DS, Patel S, Feiner L (2013). Long-term outcomes of ranibizumab therapy for diabetic macular edema: the 36-month results from two phase III trials: RISE and RIDE. Ophthalmology.

[10] Menchini U, Bandello F, De Angelis V, Ricci F, Bonavia L, Viola F (2015). Ranibizumab for Visual Impairment due to Diabetic Macular Edema: Real-World Evidence in the Italian Population (PRIDE Study). J Ophthalmol.

[11] Amissah-Arthur KN, Panneerselvam S, Narendran N, Yang YC (2012). Optical coherence tomography changes before the development of choroidal neovascularization in second eyes of patients with bilateral wet macular degeneration. Eye Lond Engl.

[12] Zarranz-Ventura J, Liew G, Johnston RL, Xing W, Akerele T, Mckibbin M (2014). The neovascular age-related macular degeneration database: report 2: incidence, management, and visual outcomes of second treated eyes. Ophthalmology.

[13] Green-Simms AE, Ekdawi NS, Bakri SJ (2011). Survey of intravitreal injection techniques among retinal specialists in the United States. Am J Ophthalmol.

[14] Age-Related Eye Disease Study Research Group (2001). A randomized, placebo-controlled, clinical trial of high-dose supplementation with vitamins C and E and beta carotene for age-related cataract and vision loss: AREDS report no. 9. Arch Ophthalmol Chic Ill 1960.

[15] Maguire MG, Daniel E, Shah AR, Grunwald JE, Hagstrom SA, Avery RL (2013). Incidence of choroidal neovascularization in the fellow eye in the comparison of age-related macular degeneration treatments trials. Ophthalmology.

[16] Barbazetto IA, Saroj N, Shapiro H, Wong P, Ho AC, Freund KB (2010). Incidence of new choroidal neovascularization in fellow eyes of patients treated in the MARINA and ANCHOR trials. Am J Ophthalmol.

[17] Wang JJ, Rochtchina E, Lee AJ, Chia E-M, Smith W, Cumming RG (2007). Ten-year incidence and progression of age-related maculopathy: the blue Mountains Eye Study. Ophthalmology.

[18] Clemons TE, Milton RC, Klein R, Seddon JM, Ferris FL, Age-Related Eye Disease Study Research Group (2005). Risk factors for the incidence of Advanced Age-Related Macular Degeneration in the Age-Related Eye Disease Study (AREDS) AREDS report no. 19. Ophthalmology.

[19] Barthelmes D, Walton RJ, Arnold JJ, McAllister IL, Simpson JM, Campain A (2014). Intravitreal therapy in bilateral neovascular age-related macular degeneration. Ophthalmology.

[20] Gonder JR, Walker VM, Barbeau M, Zaour N, Zachau BH, Hartje JR (2014). Costs and Quality of Life in Diabetic Macular Edema: Canadian Burden of Diabetic Macular Edema Observational Study (C-REALITY). J Ophthalmol.

[21] Mitchell P, Annemans L, Gallagher M, Hasan R, Thomas S, Gairy K (2012). Cost-effectiveness of ranibizumab in treatment of diabetic macular oedema (DME) causing visual impairment: evidence from the RESTORE trial. Br J Ophthalmol.

[22] Solomon SD, Jefferys JL, Hawkins BS, Bressler NM, Submacular Surgery Trials Research Group (2007). Incident choroidal neovascularization in fellow eyes of patients with unilateral subfoveal choroidal neovascularization secondary to age-related macular degeneration: SST report No. 20 from the Submacular Surgery Trials Research Group. Arch Ophthalmol.

[23] Nuzzi R, Tridico F (2015). Local and systemic complications after intravitreal administration of anti-vascular endothelial growth factor agents in the treatment of different ocular diseases: a 5-year retrospective study. Semin Ophthalmol.

[24] Bakri SJ, Risco M, Edwards AO, Pulido JS (2009). Bilateral simultaneous intravitreal injections in the office setting. Am J Ophthalmol.

[25] Abu-Yaghi NE, Shokry AN, Abu-Sbeit RH (2014). Bilateral same-session intravitreal injections of anti-vascular endothelial growth factors. Int J Ophthalmol.

[26] Lima LH, Zweifel SA, Engelbert M, Sorenson JA, Slakter JS, Cooney MJ (2009). Evaluation of safety for bilateral same-day intravitreal injections of antivascular endothelial growth factor therapy. Retina Phila Pa.

[27] Martin DF, Maguire MG, Ying G, Grunwald JE, Fine SL, CATT Research Group (2011). Ranibizumab and bevacizumab for neovascular age-related macular degeneration. N Engl J Med.

[28] Davis RP, Schefler AC, Murray TG (2010). Concomitant bilateral intravitreal anti-VEGF injections for the treatment of exudative age-related macular degeneration. Clin Ophthalmol Auckl NZ.

[29] Mahajan VB, Elkins KA, Russell SR, Boldt HC, Gehrs KM, Weingeist TA (2011). Bilateral intravitreal injection of antivascular endothelial growth factor therapy. Retina Phila Pa.

